# The Good Schools Toolkit to prevent violence against children in Ugandan primary schools: study protocol for a cluster randomised controlled trial

**DOI:** 10.1186/1745-6215-14-232

**Published:** 2013-07-24

**Authors:** Karen M Devries, Elizabeth Allen, Jennifer C Child, Eddy Walakira, Jenny Parkes, Diana Elbourne, Charlotte Watts, Dipak Naker

**Affiliations:** 1 London School of Hygiene and Tropical Medicine, 15-17 Tavistock Place, London WC1H 9SH, UK; 2 Institute of Education, London, UK; 3 Makerere University, Kampala, Uganda; 4 Raising Voices, 16 Tufnell Drive, P O Box 6770, Kampala, Uganda

**Keywords:** Corporal punishment, Primary school, Violence, Uganda, Mental health, Education

## Abstract

**Background:**

We aim to evaluate the effectiveness of the Good School Toolkit, developed by Raising Voices, in preventing violence against children attending school and in improving child mental health and educational outcomes.

**Methods/design:**

We are conducting a two-arm cluster randomised controlled trial with parallel assignment in Luwero District, Uganda. We will also conduct a qualitative study, a process evaluation and an economic evaluation. A total of 42 schools, representative of Luwero District, Uganda, were allocated to receive the Toolkit plus implementation support, or were allocated to a wait-list control condition. Our main analysis will involve a cross-sectional comparison of the prevalence of past-week violence from school staff as reported by children in intervention and control primary schools at follow-up.

At least 60 children per school and all school staff members will be interviewed at follow-up. Data collection involves a combination of mobile phone-based, interviewer-completed questionnaires and paper-and-pen educational tests. Survey instruments include the ISPCAN Child Abuse Screening Tools to assess experiences of violence; the Strengths and Difficulties Questionnaire to measure symptoms of common childhood mental disorders; and word recognition, reading comprehension, spelling, arithmetic and sustained attention tests adapted from an intervention trial in Kenya.

**Discussion:**

To our knowledge, this is the first study to rigorously investigate the effects of any intervention to prevent violence from school staff to children in primary school in a low-income setting. We hope the results will be informative across the African region and in other settings.

**Trial registration:**

clinicaltrials.gov NCT01678846

## Background

Violence against children in schools is common practice in many low-, middle- and high- income countries, and research into prevention and treatment has been outlined as a priority in the World Report on Violence against Children [1]. Children spend more time at school than anywhere else other than their family home, and can suffer violence from other children, teachers and other school staff. Fear, anxiety and injuries caused by violence may play a large role in both children’s absenteeism and low educational achievement, thus affecting progress towards achieving the Millennium Development Goals. Despite this, in most countries evidence is lacking from rigorously conducted studies on the prevalence, epidemiology and consequences of violence against children in school. Even fewer studies have investigated how to prevent such violence against children.

What data do exist indicate a high prevalence of physical corporal punishment by teachers in African schools. One study of secondary schools in Alexandria, Egypt, found that nearly 80% of boys and 62% of girls incurred physical punishments, such as from being hit with hands, sticks, straps, shoes, and from being kicked [2]. Primary school teacher reports from Illorin, Nigeria, indicate that 80% had observed pupils being disciplined with a cane. Of these, 20% of the teachers report having observed students being hit on the head and face [3]. In Uganda, no rigorous, representative prevalence data exist, but anecdotal reports and an nongovernmental organization (NGO) survey indicate more than 80% of children have experienced physical punishments such as caning and slapping by teachers [4]. This high prevalence is likely due at least in part to widespread norms condoning the use of physical discipline to punish children, among parents, teachers and community members [4]. More research exists on sexual violence in schools suffered by girls in Africa [5,6], and qualitative reports indicate girls in Ugandan secondary schools report sexual violence and harassment from teachers and fellow students, and not being able to report it for fear of reprisals [7].

Experience of violence is a well-known risk factor for ill health and for poorer educational outcomes. Those who have experienced violence as children, for example, childhood sexual abuse, are at increased risk for depression [8,9], suicide [10], risky sexual behaviour [11], and increased alcohol consumption [12]. Children who experience severe physical violence are at increased risk of adverse mental health outcomes, injury and disruptive behaviour [13]. Studies have shown that abused children are at increased risk for developing conduct disorders [14], which predict later use of violence in adult relationships [15]. Experience of childhood physical and sexual abuse also longitudinally predicts poor performance on school tests [16]. Children who experience violence from other students in school may be more likely to miss classes and to drop out, which directly affects their educational performance and life trajectory [1].

### Prevention

Systematic reviews of interventions to prevent violence in schools provide an overview of existing research, which has largely been focused on childhood sexual abuse [17,18], bullying [19,20] and other violence between students [21]. Studies have overwhelmingly been conducted in the United States where physical violence from teachers to students is less common. One of the few studies conducted outside North America that addressed teachers’ discipline practices included very young children attending community preschools in Jamaica. This study tested the Incredible Years curriculum for teachers, which provides instruction in teaching techniques and alternative discipline strategies. Observers in the pilot study recorded large improvements in teachers’ management of classes, discipline techniques and children’s prosocial behaviour in the intervention versus control preschools [22].

There is a high level of interest among international NGOs, policy makers and governments both in creating child-friendly schools [23] and in improving the quality of education in schools. International mental health experts have also called for bullying and school violence to be addressed to improve child mental health in low- and middle-income countries [24]. Several programmatic initiatives are underway [23], yet there is a paucity of data and interventions that have been tested to reduce school-based violence against children.

### Aims and objectives

We aim to conduct an evaluation of the Good School Toolkit, which is designed to prevent violence against children in schools and to improve the quality of education. The program provides information about alternative discipline techniques and teaching techniques, and employs standard behavioural change strategies (such as developing a goal, making an action plan to achieve it, and monitoring and rewarding progress) with various actors within the school setting to change violent behaviour. As highlighted previously, available data suggest that corporal punishment by teachers is a common form of violence that children experience in Ugandan primary schools. Corporal punishment is the main form of violence addressed in the Good School Toolkit. The primary objective of the Good Schools Study, therefore, is to assess the impact of the Good Schools Toolkit on children’s experiences of violence by school staff among those attending school in Luwero District, Uganda. Secondary objectives are to assess the impact of the toolkit on child mental health and educational outcomes.

## Methods/design

### Design

**Figure 1:**
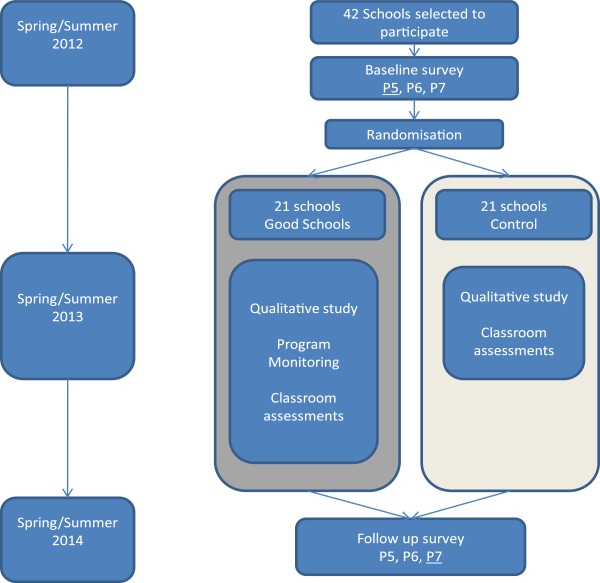
Trial timeline.

The Good Schools Study consists of a two-arm cluster randomised controlled trial, an embedded qualitative study, a process evaluation and an economic evaluation. The study is a partnership between the London School of Hygiene and Tropical Medicine, Raising Voices, Makerere University, and the Institute of Education. This publication describes the design of the trial component, which will measure the impact of the Good School Toolkit intervention. The trial will involve two cross-sectional surveys, one at baseline and one at endline (Figure 1). Our main analysis will involve a cross-sectional comparison of end line data; here we report procedures for our end line survey. The same procedures were used at baseline.

### Intervention

The experimental group is receiving the Good School Toolkit (http://raisingvoices.org/download-good-school-toolkit/) and implementation support over an 18-month period. The Toolkit is an established intervention, which has been popular in Uganda since it was developed 6 years ago by Raising Voices. This implementation period was chosen based on Raising Voices programmatic experience and is the period during which the Toolkit is designed to produce changes in a school.

The Toolkit is designed to be implemented with minimal cost, appropriate for low-resource settings. Development was in close collaboration with six Ugandan schools over 18 months, and the Toolkit has had two rounds of extensive revisions based on in-depth feedback from 40 schools to increase acceptability and effectiveness. Raising Voices also conducted interviews with 200 teachers, and 91% of those teachers reported that both teachers and students in their schools were using the materials, and nearly 100% reported that the materials were useful for their school. The Toolkit is currently being used in approximately 450 Ugandan schools, and in all of these cases, schools or their NGO partners have sought the Toolkit from Raising Voices, thereby showing demand. No schools that have been offered the Toolkit have declined to use it.

The intervention content is based on well-established behavioural change techniques that have been shown to be effective in a variety of fields [25] and have been included in interventions that reduce intimate partner violence perpetration [26] and change teacher behaviour and discipline methods in primary schools [22,27]. Drawing on the Transtheoretical Model [28], the Toolkit uses a six-step process to engage teachers, students, administration, and parents to reflect on how they can promote quality of education in their school. The intervention support materials consist of booklets, posters and facilitation guides for 60 different activities. These activities are related to creating a better learning environment, to respecting each other, to understanding power relationships, to using non-violent discipline, and to improving teaching techniques.

The process begins by selection of a school-based ‘protagonists’, usually two motivated members of staff and two students in each school, to engage other staff, students, and the administration to set school-wide goals [25] and to develop action plans [25] with specific dates for deliverables [25]. Activities are facilitated by the protagonist and other school personnel, and use written materials to encourage empathy by facilitating reflection [25] on experiences of violence [26], to provide new knowledge [25-27] on alternative non-violent discipline, and to provide opportunities to practice new behavioural skills [25-27]. Students are also encouraged to reflect on use of physical, sexual and emotional violence in relationships with each other. Schools are encouraged to self-monitor [25] their progress according to their action plans on a termly basis, and are prompted [25] to do so initially by Raising Voices. Reinforcement of new information and ideas [25,26] feedback on progress and modeling of new techniques and behaviours [25,27] is provided by visits from the Raising Voices team, and also within school by ‘protagonists’ to their peers as they gain new knowledge and skills. Schools are encouraged to reward [25] successful achievement of their goals and action plan deliverables by creating celebrations. Because the intervention engages multiple groups within a school (teachers, administration, students, and also parents), changing ideas and attitudes in different groups also creates social support [25-27] for behavioral change as the intervention progresses. Social support and specific techniques, tips, and experiences are also provided by a ‘Peer Learning Network’ of more than 100 schools using the Toolkit, moderated by Raising Voices.

### Control

If the intervention is shown by the trial to be effective, control schools will be offered the following package after the end of the study: the Good Schools Toolkit, an introductory session to support implementation, and access to a peer learning support network to help them support implementation of the Toolkit. During the study, the control schools will not receive any form of programming; however, they will have the same schedule of survey, class and school level assessments as the intervention schools. If the intervention is shown by the trial to be ineffective, this will be explained to control schools. Raising Voices will refine and update the content of its programming if this is the case, and updated materials will instead be offered to control schools.

### Setting

The study is being conducted in Luwero District, Uganda, which has both rural and more urban areas. Luwero has an estimated 433,100 people, 60% of whom are aged below 18 years. Luwero is demographically similar to the rest of Uganda, with an equal sex ratio of males to females and a mean household size of 4.4 persons. A total of 77% of the population aged 10 years or over was literate in 2002 (versus 68% nationally), 72% had access to safe water (versus 61% nationally), and 7.2% had access to electricity (versus 8% nationally). Both in Luwero district and at a national level, subsistence farming methods were used by 68% of households, and the majority of households (97%) relied on firewood and charcoal for cooking.

Luwero District was chosen because it is within the catchment areas of the implementing partners for this study. There are currently no prevalence or epidemiological data available on children’s experiences of violence or independent data on educational performance. Our study will provide new information for the District.

### Participant selection and inclusion criteria

A two-stage selection process has been employed. In the first stage, schools were randomly selected to participate in the trial. In the second stage, within each school, all individual staff members and a random sample of Primary 5, 6 and 7 students will be invited to participate in the follow-up survey.

#### Schools

Our implementing partners are able to provide support to a maximum of 21 schools; with the inclusion of 21 control schools the total sample size is 42 schools. These were chosen to minimise selection bias as far as possible and to represent larger schools in Luwero. Using the official 2010 list of all 276 primary schools in Luwero as our sampling frame, we excluded 105 very small schools (with fewer than 40 registered Primary 5 students) and 20 schools with existing governance interventions, and then stratified the remaining 151 schools by the gender ratio of their pupils, into >60% girls, mixed, or >60% boys. From these 151 schools, we selected a random sample of 42 schools, proportional to size of the stratum. Of these schools, 100% agreed to participate in the study. Up to date lists of all Primary 5, 6 and 7 students, and a list of all teaching and non-teaching staff will be obtained from school head teachers at endline.

#### Individual participants

From these class lists, up to 130 children will be randomly selected for individual interviews at follow-up, with the aim of successfully interviewing at least 60 children per school. If a school has fewer than 130 students in P5 to 7, all children will be invited for interview. We chose to focus on students in P5 to 7 students, aged approximately 10 to 14 years, because they would be better able to respond to survey questions about their experiences versus younger children. All school staff will be invited for an interview. Research teams will be in each school for 3 to 6 days; at least one repeat visit will be made to find students who have been absent for that entire period. Students deemed unable to understand the study consent form (who will be therefore unable to provide informed consent) will be excluded.

### Sensitisation and recruitment

During the planning stages of the study, Raising Voices staff visited Ministry of Education and Sports officials at national and district levels. At the national level, support for the study was indicated by the Ministry Of Education And Sports. At the district level meetings have been held with the District Education Officer in Luwero, who has given permission for the study to take place.

Invitations to individual schools selected for participation were issued by letter from Raising Voices to school Head Teachers. These letters were followed by a visit from senior program officers from Raising Voices to explain further details about what would be involved in participating in the study. Consent for participation was sought from head teachers; of these head teachers, 100% agreed to participate.

Consent for classroom level assessments, which will involve observations of whole classes, will also be sought from head teachers on the day of the assessment. At follow-up, all staff in participating schools will be invited to participate in individual level data collection by providing informed consent. An opt-out consent strategy will be used for students, consistent with other school-based research on sensitive topics in Uganda [29]. Same as at baseline, parents of children in participating schools will be informed about the study in several different ways and advised that they can opt their children out from participating. Information meetings will be held at each participating school with a staff member from school administration and a representative from the study team to explain the study to community leaders, and parents and guardians of children. These meetings will emphasize that participation of children in research data collection is voluntary, that they have the opportunity to opt out of the study at any time, and not to answer any questions that they do not want to. It will also be made clear that the study does not involve collecting of any biological specimens, and involves only asking questions of children and administering standard educational tests. Parents and community leaders will be asked to circulate word about the study to others in their community.

In participating schools, each P5, P6, and P7 child will also receive a written notice to carry home to his or her parents or caregivers. At baseline, identical procedures were used, and all of these meetings and notices were held before the baseline survey and allocation took place; schools, parents, or children were not aware of whether or not their school would receive the intervention when they completed the survey.

Individual students selected to participate in the survey will be approached within their school, and informed consent will be sought. The consent form will be read aloud to each child. This form contains a description of study procedures and will remind children that they do not have to participate and have the right to stop the interview at any time. Children will also be informed that if an interviewer feels a child’s safety is at risk, the interviewer is obligated to discuss the case with the District Probation Officer or with another party responsible for child protection locally.

### Outcomes

We aim to determine the impact of the Good School Toolkit on violence, well-being and educational outcomes in children attending primary school. Specifically, we will:

1. Examine whether there is a difference in children’s self-reported experience of past week physical violence by school staff, in schools that receive the intervention versus those that do not (primary outcome);

2. Examine impacts of the intervention on children’s educational achievement (word recognition and reading comprehension in Luganda and English, spelling, and written numeracy), and children’s mental health (symptoms of common mental disorders and self-reported feelings of safety and well-being in school) (secondary outcomes).

### Statistical power

Preliminary analysis of baseline data has shown a prevalence of past week physical violence (primary outcome) of approximately 50%, and an estimated intracluster correlation coefficient of 0.06. If we see similar levels at follow-up, allowing for a possible loss of two schools per arm and a conservative estimate of 60 pupils per school, we will be able to detect a reduction of approximately 13% in the prevalence of reported violence in the schools receiving the intervention, with 80% power and a 5% level of significance. One other pilot trial we are aware of that evaluated an educational and behavioural change program observed a more then 50% reduction in ‘negative teacher behaviours’ as rated by classroom observers over the trial period [22], so we should be well placed to detect effects of the Good School Toolkit.

### Survey procedures

All questions related to violence and mental health will be asked during individual interviews with Luganda-speaking staff who receive 3 weeks of in-depth training. Individual interviews will be done using a questionnaire programmed into mobile phones (completed by an interviewer). Some educational assessments will be done on paper in groups.

### Survey instruments

Students’ experiences of violence will be assessed using behaviourally specific questions about acts of violence adapted from the IPSCAN Child Abuse Screening Tool (ICAST) [30], and the WHO Multi Country Study on Women’s Health and Domestic Violence against Women (WHO MCS) [31]. We will assess acts of different severity levels and timeframes (past week, frequency in past year, and before past year). The main focus will be on violence by school staff, but we will also ask about violence from other perpetrators. Initial items were reviewed by a panel of teachers and Raising Voices staff to ensure that they would reflect the experiences of primary school children in the Ugandan context. The instrument was then pilot tested with children in Kampala and refined based on this feedback, and will be further refined based on the results of the baseline survey.

Children will be administered literacy, numeracy and sustained attention assessments adapted from an intervention study in schools in Kenya, involving one of the study team (Dr Allen) [32]. The Strengths and Difficulties Questionnaire [33] brief screening instrument will be used to measure symptoms of common childhood mental disorders, including depression, anxiety, and conduct disorder. Items measuring children’s attitudes to corporal punishment developed by the research team will also be included.

School staff will be asked about perceptions of student discipline and learning needs, attitudes about corporal punishment and violence, as well as their teaching methods, participation in school policy development and culture. Experiences of violence will be queried, using items from the WHO MCS [31]. Mental health and well-being will be assessed using the Self Report Questionnaire-20 [31], and items adapted from the ICAST (as above) will be used to ask staff about their use of violence toward students in the past week, and for other time frames.

### Confidentiality and data management

Interviews will be conducted in open spaces, where interviewer-participant pairs are out of earshot but within sight of others, to protect confidentiality and ensure child safety. All data collected on mobile phones (all violence and mental health data) will be identified only by ID number, for both staff and students. All data collected by mobile phone will be stored on a password protected databases that will be online-accessible only to senior study personnel, and backed-up daily on a password-protected laptop. Students will write their names on standard educational tests, which will be double marked and double entered into a customised database, separate from other sensitive data.

### Randomisation

Schools were allocated to receive either the intervention or a wait-list control at a public meeting with all school head teachers before the start of the 2012 September school term. Stratified randomisation was used to ensure balance in regard to key factors (baseline violence, whether the school was urban or rural, and a qualitative assessment of the likelihood of attrition over the course of the trial).

### Analysis

Primary analysis will be carried out based upon the groups as randomised (‘intention to treat’). Results will be presented as appropriate effects sizes with a measure of precision (95% confidence intervals). Clustering by school will be allowed for in all analyses. Our main analysis of the primary outcome, the prevalence of past week experience of violence, and secondary mental health and educational outcomes will be cross-sectional analyses comparing the prevalence at follow-up between the two arms of the trial. In cross-sectional analyses, baseline school level summaries will be adjusted for, where appropriate [34]. Additional analyses will use longitudinal data and compare the change in individual scores over time between intervention and control schools. In longitudinal analyses, baseline measures of the outcomes will be adjusted for, along with other covariates such as gender and baseline age.

### Trial organisation, governance, and adverse events

The trial is overseen by the principal investigator (KD) and run by a dedicated manager (JC). A data manager is responsible for monitoring data quality at baseline and follow-up data collection points, and for interim data collection on the intervention implementation. The main data monitoring concerns for a trial of this nature relate to ensuring that cases where children have been exposed to severe forms of violence are detected and that adequate steps are taken to protect them. Comprehensive referral plans have been developed for baseline and follow-up data collection (described in Ethical considerations). We will also collect ongoing monitoring data from schools during the implementation process; any child protection concerns that arise during the implementation period will proceed through the same referral pathway.

The intervention under study is behavioural and we do not anticipate adverse events occurring as a result of the intervention itself. However we will collect interim qualitative data from representatives of school staff and also from representatives of student committees about unexpected consequences and any adverse consequences of the intervention that have become apparent during implementation. This data will be collected by telephone and in-person interviews by the trial monitoring data officer, and will be entered into a computer on a continuous basis. Any adverse events will be reported immediately to the study manager (JC), the principal investigator (KD), and the implementing partners (DN). This committee will decide upon and enact appropriate responses.

### Ethical considerations

In any research on violence against children, ethical considerations and child protection are paramount. We developed comprehensive protocols to ensure that children are protected and referred as necessary over the course of the study, and have received full ethical approvals from both the LSHTM (#6183) and the Uganda National Council for Science and Technology (SS 2520).

Our referral strategy for children in need of support involves working within existing child protection structures for the Ministry of Education; the health, legal, and community welfare sectors, with additional support from local NGOs; and implementing study partners to ensure proper procedures are followed. In brief, all children who participate in the study, regardless of what they disclose, will be offered the opportunity to visit with a trained counsellor who is fluent in Luganda. For children who disclose more severe experiences of violence in the past week or past year, the District Probation Officer and the representative from our local Luwero partner NGO, will be informed, and will refer cases onward in accordance with local policy. Children who disclose recent sexual violence, severe physical violence, or injury, or who have otherwise urgent conditions, will be taken immediately to a health centre, and the District Probation officer and local Luwero partner NGO representative will be informed so that further follow-up may take place.

The nature of case management and follow-up provided by the existing child protection system is the subject of a nested study within the larger Good Schools Study.

## Discussion

To our knowledge, this is the first study to rigorously investigate the effects of any intervention to prevent violence from school staff to children in primary school in a low income setting. We hope the results will be informative across the African region and in other settings.

Our study design has some strengths and limitations. The site for implementation of the Toolkit for this evaluation was chosen because it is within the catchment areas for our implementing NGO partners, in the same way that an area for implementation would normally have been chosen. Luwero is typical of Uganda in many socio-demographic ways. We have selected schools to be representative of larger schools in Luwero District, thus representing the majority of pupils in the District. Because of this selection procedure, our results will be generalisable to larger schools in Luwero. We intend to use qualitative and quantitative methods to identify further factors related to uptake of the intervention, and to generate lessons about generalisability to the rest of Uganda and beyond. We will focus at the level of individual schools and beyond (for example, identifying key officials at the National or District level who facilitate uptake, or identifying specific policies in place in certain subcounties that affect uptake).

Our main analysis involves a cross-sectional comparison of the prevalence of past-week experience of physical violence by school staff as reported by children. We chose this design, rather than following individual students longitudinally, for several reasons. One, the Good School Toolkit is designed to work at the level of the school, and produce changes not only individuals but in school structures and governance. Thus, we aimed to primarily evaluate impact at the school level. Two, in primary school populations in Uganda, particularly towards the end of Primary school in P7, there is substantial attrition of students over time. The repeated cross-sectional design alleviates potential selection bias introduced by attrition of students between rounds of the survey.

In violence research, gold-standard measures are self-reports of behaviourally specific acts; however it is likely that there will be some under-reporting because of the trauma and stigma associated with abuse [31]. As a result of the intervention, violence may be considered less normative and reporting of violence may increase in the intervention schools. This effect will be in the opposite direction of the intervention effect, making this trial a conservative test of intervention impact. We will also have the opportunity to quantitatively explore under-reporting at baseline versus follow-up using data from children who have been referred as a result of experiencing violence. We will be able to compare case notes documenting what these children have disclosed to counsellors versus what they have reported in the survey.

It is possible that some spillover may occur during the trial, which could lead to underestimation of intervention effects. However, this trial is designed as a pragmatic trial, and will produce estimates of the effectiveness of the Good School Toolkit under real-world programmatic conditions, rather than ideal or artificial experimental conditions. The Good Schools materials are publicly available on the Raising Voices website. It is also likely that some school staff will migrate during the course of the trial, and it is possible that some staff from intervention schools will be placed in control schools. However, only intervention schools will receive implementation support on an ongoing basis, which we think will be instrumental to intervention success. School staff will be questioned about any previous exposure to the Good Schools materials, and school-level assessments will query whether other programs similar to Good Schools have been implemented. We will include an analysis of intervention effects by level of exposure in our study outputs.

Our study is one of the first trials of a violence prevention program in schools in a low-income setting. The Good School Toolkit is designed to be a low-cost method of intervention, appropriate for wide scale-up in low and middle income settings. The results of this study are awaited by the Uganda Ministry of Education and Sports, and will be of interest to other governments, donors and policy-makers within and outside the East African region.

## Trial status

The trial is ongoing, and recruitment of individual participants for the follow-up survey is scheduled to start in June 2014 and to be complete in July 2014.

## Abbreviations

ICAST: IPSCAN child abuse screening tool; WHO MCS: WHO Multi Country Study on Women’s Health and Domestic Violence against Women.

## Competing interests

Dipak Naker is co-Director of Raising Voices, and developed the Good School Toolkit.

## Authors’ contributions

KD designed the study, supervised data collection and drafted the manuscript. EA participated in the design of the study and performed statistical analysis. JC managed the fieldwork for the baseline survey and participated in the design of some aspects of the study. CW, JP, EW and DE participated in the design of the study. DN developed the intervention, initiated the idea to do a study, contributed to the design of the study, and obtained funding for the baseline research. All authors read and approved the final manuscript.
